# Increased enslaving in elderly is associated with changes in neural control of the extrinsic finger muscles

**DOI:** 10.1007/s00221-018-5219-1

**Published:** 2018-03-23

**Authors:** M. Mirakhorlo, H. Maas, H. E. J. Veeger

**Affiliations:** 1 Department of Human Movement Sciences, Faculty of Behavioral and Movement Sciences, Vrije Universiteit Amsterdam, Amsterdam Movement Sciences, Van der Boechorststraat 7, 1081 BT Amsterdam, The Netherlands; 2 Department of Biomechanical Engineering, Delft University of Technology, Delft, The Netherlands

**Keywords:** Hand, Finger, Aging, Motor control, EMG

## Abstract

Aging has consequences for hand motor control, among others affecting finger force enslaving during static pressing tasks. The aim of this study was to assess whether the extent of finger force enslaving changes with aging during a task that involves both static and dynamic phases. Ten right-handed young (22–30 years) and ten elderly subjects (67–79 years) were instructed to first exert a constant force (static phase) and then flex their index finger while counteracting constant resistance forces orthogonal to their fingertips (dynamic phase). The other fingers (non-instructed) were held in extension. EMG activities of the flexor digitorum superficialis (FDS) and extensor digitorum (ED) muscles in the regions corresponding to the index, middle and ring fingers together with their forces and position of index finger were measured. In both elderly and young, forces exerted by the non-instructed fingers increased (around 0.6 N for both young and elderly) during isotonic flexion of the index finger, but with a different delay of on average 100 ± 72 ms in elderly and 334 ± 101 ms in young subjects. Results also suggest different responses in activity of FDS and ED muscle regions of the non-instructed fingers to index finger flexion between elderly and young subjects. The enslaving effect was significantly higher in elderly than in young subjects both in the static (12% more) and dynamic (14% more) phases. These differences in enslaving can at least partly be explained by changes in neuromuscular control.

## Introduction

Aging generally has a detrimental effect on hand and wrist function, such as a lower accuracy in finger force production and a deterioration in grip strength (Ranganathan et al. 2001; Vieluf et al. 2013). In terms of hand motor control, aging has been found to diminish enslaving (Oliveira et al. 2008; Shinohara et al. 2003a, b), which is defined as the inability of fingers to produce force and/or move independently (Lang and Schieber 2004; Zatsiorsky et al. 2000). There are two types of factors that can influence the extent of finger enslaving. First, mechanical factors including linkages between the tendons of extrinsic flexor muscles (Frohse and Frankel 1908) and myofascial connections between adjacent muscle bellies (Maas and Sandercock 2010). Second, neural factors including shared motor units in extrinsic finger muscles and overlap of motor cortex areas controlling different fingers (van Duinen and Gandevia 2011; Zatsiorsky et al. 2000).

In young healthy subjects, studies on enslaving mostly focused either on static finger pressing (Sanei and Keir 2013; Zatsiorsky et al. 2000) or on finger movement tasks (Häger-Ross and Schieber 2000; Kim et al. 2008; Lang and Schieber 2004). In studies on static finger pressing tasks, neural factors were considered as the predominant cause for the observed enslaving effects (Latash et al. 2002; Zatsiorsky et al. 2000). This conclusion was based on finding similar enslaving effects when pressing with distal and proximal phalanges. Forces exerted by proximal phalanges were assumed to be produced by intrinsic muscles, which do not appear to be mechanically inter-connected. Kim et al. (2008) investigated the effects of movement velocity on finger force enslaving during a free flexion/extension task. They hypothesized that, in case of mechanical interaction dominance in enslaving, transmitted forces and thus enslaving should be velocity-dependent. Although they did not find effects of velocity on the extent of enslaving, some viscous properties were observed in the system. Therefore, an unequivocal conclusion could not be drawn. In contrast to these studies, a previous study showed that the enslaving effect was similar for active and passive movements suggesting a major role for mechanical connections (Lang and Schieber 2004). Involving finger movement in the task can provide insight into the mechanisms of enslaving; more exactly the role of the above described mechanical connections. These connections may be slack during finger pressing tasks, and thus not capable to transmit forces. During dynamic tasks, however, they may experience more strain and, hence, transmit forces. Thus, the type of task may determine which factors are the main cause of enslaving. Recently, an experiment was performed by our group, in which young subjects were instructed to flex their index finger while overcoming a constant sub-maximal resistance force (Mirakhorlo et al. 2017). Obtained results indicated that connective tissue linkages are (at least partly) responsible in limiting finger independency.

Age-related changes of muscle and tendon involve an increase of the amount of connective tissues in skeletal muscle (Zimmerman et al. 1993), loss of muscle mass (Goodpaster et al. 2006) and reduction in tendon compliance (Tuite et al. 1997). In terms of neural control, higher co-activation of muscles in elderly subjects compared to younger subjects (Burnett et al. 2000; Seidler-Dobrin et al. 1998) and enlarged motor units (Reviewed in (Larsson and Ansved 1995)) have been reported. All these aspects might lead to higher enslaving effects with increasing age. Up till now, enslaving in elderly subjects was mostly investigated for static pressing tasks (Kapur et al. 2010; Olafsdottir et al. 2008; Oliveira et al. 2008; Shinohara et al. 2003a, b) and only study for a free finger movement task (Van Beek et al. 2017). Shinohara et al. (2003b) reported lower indices of finger force enslaving in the elderly when compared to young subjects. They measured finger forces during a particular static position restraining forearm and hand motion and attributed these changes to adaptations in central neural strategies rather than the alterations in mechanical interactions. In contrast, higher indices of finger movement enslaving in elderly during free movement tasks were reported recently (Van Beek et al. 2017). The higher amount of enslaving for elderly may be explained by the fact the wrist was not restrained in their study. The extrinsic muscles of the fingers have also moment arms about the wrist, which may have consequences for finger enslaving. Assessment of muscle activation patterns of extrinsic muscles may help to understand the roles of these muscles in enslaving. Van Beek et al. (2017) measured EMG of an extrinsic finger flexor and extensor. Their results revealed an activation pattern of the finger specific muscle regions that was more evenly distributed in the elderly in comparison with young subjects.

The first aim of this study was to investigate the changes with aging in the extent of finger force enslaving. Based on the changes in muscle properties, i.e. increased connective tissues (Zimmerman et al. 1993) and enlarged motor units [reviewed in Larsson and Ansved (1995)] with advancing age, higher finger enslaving was expected in the elderly. The second aim of this study was to investigate the neural control of the extrinsic finger muscles during single finger movements. Based on the results of Van Beek et al. (2017), we expected more coactivation of the finger specific muscle regions. For this purpose, we compared older subjects to young subjects of which the data were published previously (Mirakhorlo et al. 2017). We measured forces exerted at the finger tips and EMG activities of the different regions of flexor digitorum superficialis (FDS) and extensor digitorum (ED) muscles corresponding to the different fingers in response to isotonic flexion of the index finger.

## Methods

Twenty right-handed subjects participated: 10 young subjects aged between 22 and 30 and 10 elderly subjects aged between 67 and 79 years. Subjects had no (history of) neurological or peripheral disorders of the hand or wrist. None of the recruited subjects were players of musical instruments. This exclusion criterion was applied because of their higher than average degree of finger independency (Furuya et al. 2014; Rosenkranz et al. 2009). Written informed consent was obtained according to the regulations established at the Vrije Universiteit Amsterdam. The Scientific and Ethical Review Board of the department approved the experiment protocol.

### Experimental setup

**Fig. 1:**
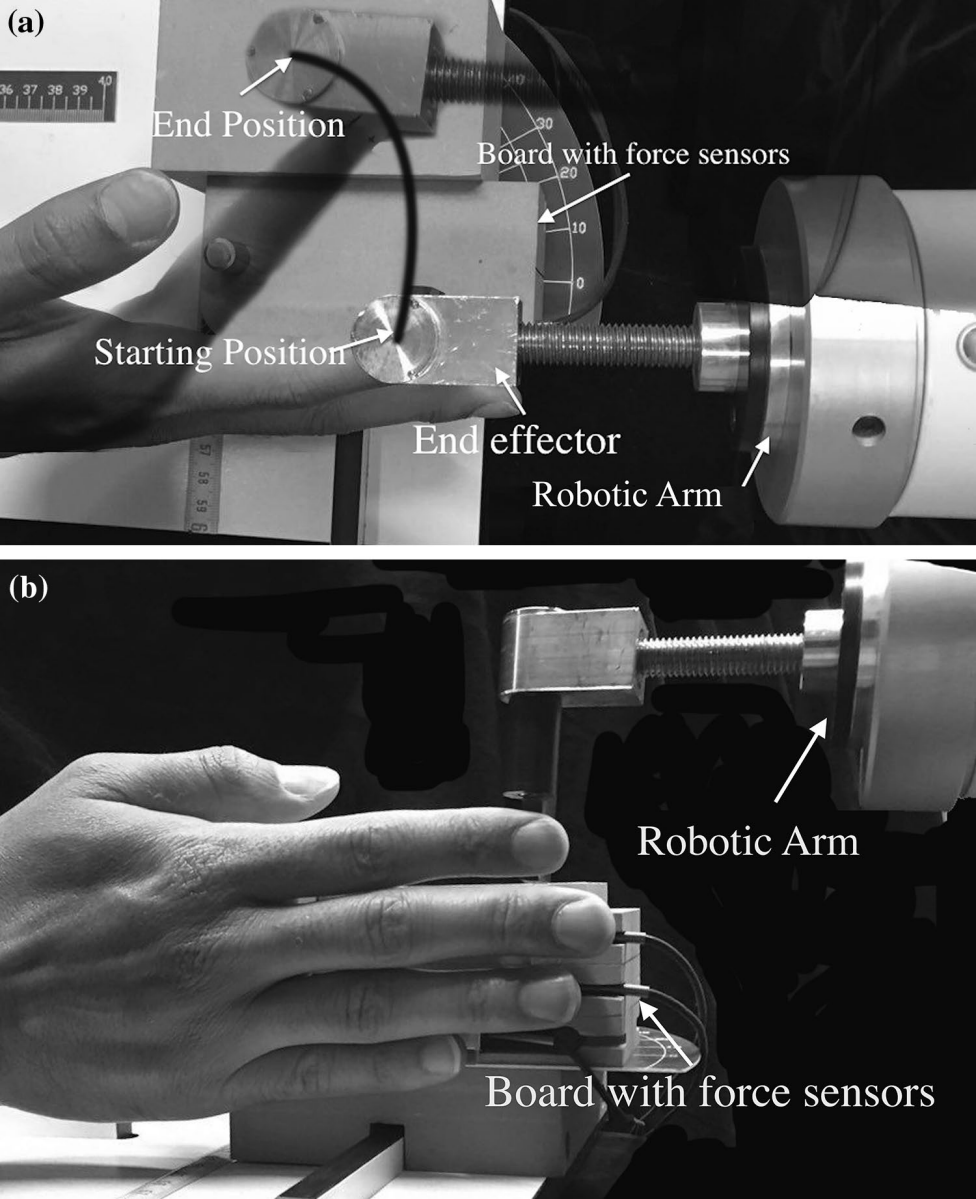
Top-view (**a**) and side view (**b**) of experimental set-up. The robotic arm, which followed the flexion movement of the index finger, and the board on which the non-instructed fingers were placed are shown. The black curved line in (**a**) is the approximate path of tip of the index finger

Subjects were requested to sit on an adjustable chair resting their forearm on a horizontal platform leaving the wrist free to move, as described in detail previously (Mirakhorlo et al. 2017). Their seating position was adjusted such that the elbow was in approximately 90° flexion. A wooden board secured to the arm rest was instrumented with three unidirectional force sensors (Futek, Irvine, USA, LSB200, maximum 5 lb). Subjects were asked to rotate their hand in a 90° pronation angle (0° is corresponding to the anatomical position) aligning their fingers with the wooden board. The position of force sensors was adjusted in two directions, along the finger and medial–lateral, such that the tips of the little, ring and middle fingers were in contact with a narrow beam (width 3 mm) at the center of the force sensors. A robotic arm (Van der Linde et al. 2002) (Haptic Master 2.2, Moog, Nieuw-Vennep, The Netherlands) equipped with a custom-made end-effector (Fig. 1) was used to provide a resistance force directed along with the trajectory of the tip of the index finger (see “Experimental protocol” below). This robotic arm measures and records the force of the index finger.

### EMG and force measurements

EMG signals were collected in a mono-polar configuration with the ground electrode placed on the ulnar styloid, amplified using a 128-channel amplifier and sampled at 2048 samples/s (Refa-136; TMSi, Oldenzaal, The Netherlands). Forces applied by the middle, ring and little finger were collected at the same sampling rate as EMGs and the amplifier (2048 samples/s, Refa-136). Electrode placement locations were shaved and cleaned with alcohol. Muscle regions of FDS and ED corresponding to the different fingers were palpated for each subject individually. Four electrodes were used for each muscle region to identify the best electrode location of each muscle region (Mirakhorlo et al. 2017). Electrodes (KendallTM H69P Cloth Electrodes, Covidien, Zaltbommel, The Netherlands) were placed on each of the regions corresponding to the index (II), middle (III) and ring (IV) fingers for both ED and FDS muscles. There were, therefore, six possible bipolar combinations for each muscle region. Muscle activities corresponding to the little finger were not measured in this study as enslaved forces (Zatsiorsky et al. 2000) or substantial EMGs for the little finger were not expected. Electrodes were linearly placed at an inter-electrode distance of approximately 2 cm. To validate the placement for each muscle region, subjects were asked to flex freely (for FDS) and extend with resistance (for ED) the related finger with real-time display of the EMG signals on a monitor.

### Experimental protocol

Subjects were asked to build up forces with their index finger (instructed finger) to the predefined level of 6 N against the fixed end-effector of the robotic arm and maintain this force for at least one second (static phase). During this phase, they were given real-time visual feedback about the applied force level on a screen in front of them. Force buildup was followed by a phase in which the end-effector was programmed to follow the path of the index finger (dynamic phase). The robotic arm was programmed such that it exerted force in the same direction as the velocity of the movement of the tip of the index finger. Subjects were asked to flex only the metacarpophalangeal (MCP) joint of the index finger from an extended position (i.e., metacarpophalangeal, MCP, proximal interphalangeal, PIP, and distal interphalangeal, DIP, joints at 0°) to a more flexed position (MCP angle at approximately 45°). The PIP and DIP joints were also free to move, but subjects were instructed to minimize this. Finger joint angles were measured and PIP and DIP joint angles were found to be changed by maximally 5° during the movement. The other, non-instructed fingers (middle, ring and little) were resting against the board with force sensors (Fig. 1). Subjects were asked to not pay attention to the non-instructed fingers. Fingertip forces were recorded simultaneously with EMG signals, as well as the end point trajectory of the index finger. The robotic arm and the EMG–force data recording system were synchronised using a start-stop pulse signal to both devices. Each subject repeated the task until at least three trials with an average end-effector speed of around 3 cm/s were recorded. This average speed was selected, as in pilot measurements it corresponded closely to the self-selected speed of finger flexion movement, which was a comfortable speed (neither too slow nor too fast) according to the feedback of the participants.

### Maximum voluntary contraction and ramp force production

To record the maximum voluntary contraction (MVC) of ED muscle regions, subjects were asked to maximally extend their finger while it was held flexed by the experimenter. MVC of the FDS muscle was recorded by asking subjects to maximally press their index finger against the fixed robotic arm while for the other fingers subjects were required to press against the board (Fig. 1). Each MVC trial was repeated three times. EMGs were averaged over three seconds during each repetition and the maximum value of the three attempts was selected for normalization.

For the different ED muscle regions (index, middle and ring finger), the electrode pair yielding the highest EMG amplitude during the MVC tests was selected. To find the best representative signal for the different FDS muscle regions (index, middle and ring finger), a ramp protocol was performed. With the index finger placed against the fixed robotic arm and the other fingers on the board (Fig. 1), subjects were asked to gradually increase flexion force to a submaximal level (around one-third of maximal force) for each finger individually during which both EMGs and forces were measured. Each subject repeated this task three times. The ratio of each FDS region and the corresponding ED muscle region was calculated for each electrode combination. The electrode combination for which the highest ratio was found during force exertion of the target finger and the lowest ratio during force exertion of the other fingers was selected for further analysis.

### Data analysis and statistics

EMG signals were high pass filtered at 20 Hz using a fifth order, zero-lag Butterworth filter. Subsequently, signals were rectified on basis of the Hilbert transformation and, then, low pass filtered at 2 Hz also using a fifth order, zero-lag Butterworth filter. Mean EMG activities and forces during the static phase, 1 s prior to the start of movement, were calculated. During the dynamic phase, EMG activities and forces at the end of the movement were used for further analysis. Force signals of all fingers and position data of the index finger were low pass filtered at 10 Hz using a third order, zero-lag Butterworth filter. Prior to the start of the experimental protocol, the fingers are not in a resting position but fully extended (Fig. 1) and exerting some force. For further analysis of force data, these forces were adjusted to zero.

**Fig. 2:**
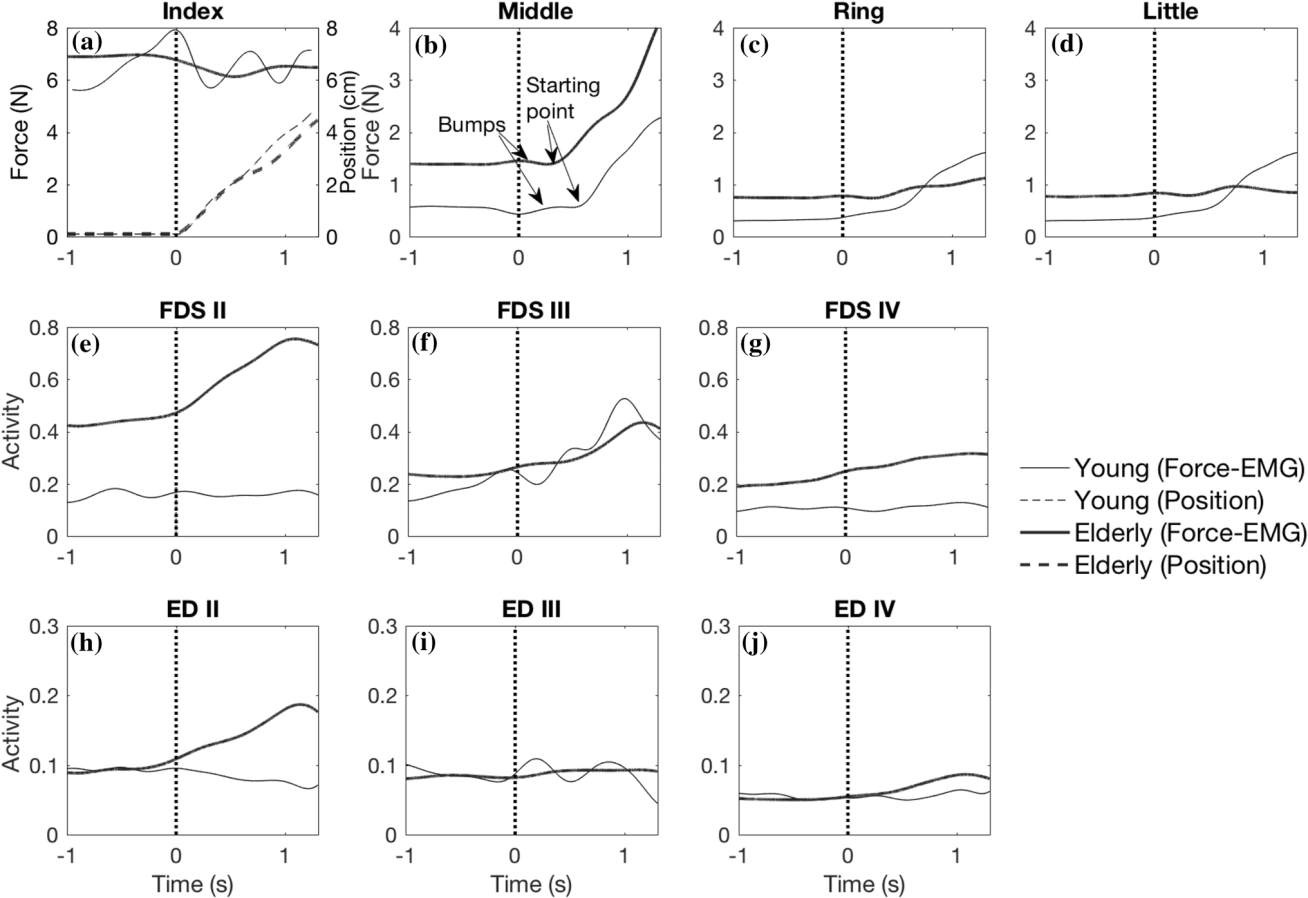
Force and EMGs of one trial of one representative elderly (black thick lines) and young (gray thin lines) subject during the static phase (time − 1 to 0 s) and dynamic phase (time 0–1.35 s). **a** Force (thick lines) and position (dashed lines) waveforms of the index finger. Forces of non-instructed fingers (**b–d**), EMGs of FDS (**e–g**) and ED muscle (**h–j**) regions for related fingers. Vertical dashed line indicates the start of index finger movement

The instant of force buildup in the non-instructed fingers was defined as an increase in force of more than 1% of the force during the static phase, the latter calculated as the mean of 1 s prior to the start of index finger movement. The time between the start of index finger movement and the instant of force buildup (the delay time) was calculated. Little bumps (small increases in force followed immediately by a force decrease) in the pattern of forces (Fig. 2b) were ignored, because they were the result of perturbations caused by the start of the movement and instability of the controller of the robotic arm [for more details see Mirakhorlo et al. (2017)]. The points that were considered to be the start of the increase in the middle finger force are indicated in Fig. 2b.

The force enslaving effect (EE) of each finger was calculated for both static and dynamic phases (see Eq. 1). For the static phase, the ratio of the averaged force exerted by the non-instructed finger to the averaged force of the index finger was used to calculate the EE. For the dynamic phase, EE was calculated as the ratio of the force exerted by the non-instructed finger at the time point when index finger movement ended, to the force of the index finger at the same time point. In addition, the difference in EE between the static and dynamic phases was calculated and named additional enslaving (ΔEE).

1 $${\text{EE}}\% =\frac{{{\text{Force}}{~_{{\text{non}} - {\text{instructed}}}}}}{{{\text{Forc}}{{\text{e}}_{{\text{index}}}}~}} \times 100\%$$

Velocity of the fingertip (robotic arm end-effector) was calculated as the first derivative of the position signal. The MCP joint flexion was estimated based on the length of the subject’s index fingers and the position of the index finger tip, assuming DIP and PIP joint angles were kept at zero degrees throughout the movement.

To compare the mean velocities of the index fingertip between old and young subjects a 2-sample *t* test was used. For the index finger, two-way repeated measures ANOVAs (within group factor: static–dynamic phase; between groups factor: young–elderly) were used to test for changes in force and EMG of FDS and ED muscles. To analyze effects on the delay times, two-way repeated measures ANOVAs (within group factor: finger, between groups: young–elderly) were used. In case of significant interactions, post-hoc *t* tests with Bonferroni correction were performed. Three-way repeated measures ANOVAs (within group factors: finger, static–dynamic phase; between groups: young–elderly) were used to test for changes in FDS, ED muscle activities, force and EE in the non-instructed fingers. In case of significant interaction between all three factors, the difference between static and dynamic of that parameter (static–dynamic change) was calculated. Two-way repeated measures ANOVAs (within group factor: finger, between groups: young–elderly) was used to analyze effects on static–dynamic changes. All analyses were performed in Matlab (2017a, Mathworks, Natick, USA). Results were considered to be statistically significant at *p* < 0.05.  

## Results

### Movement velocity of the index finger tip

There was no significant difference between young and elderly in the velocity of the tip of the index finger (*p* = 0.213). The mean velocity was 3.2 ± 0.5 cm/s for the elderly and 3.0 ± 0.3 cm/s for the young subjects.

### Delay times

**Fig. 3:**
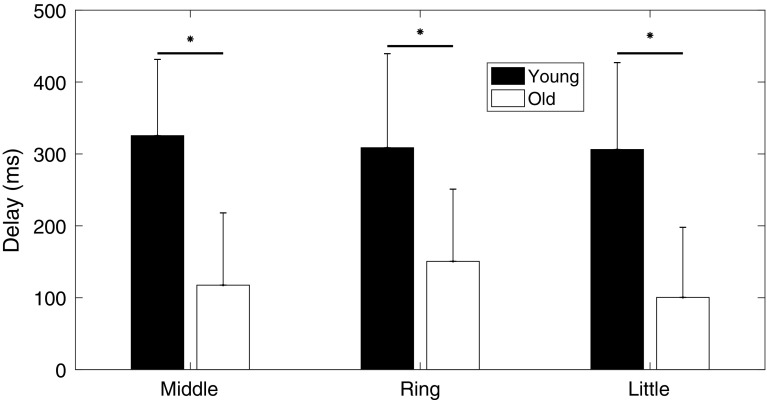
Delay times of non-instructed (middle, ring and little) fingers of young and elderly subjects. The delay time was calculated as the difference in the start of non-instructed finger force development relative to the start of instructed finger (index) position

Delay times between the start of index finger movement and force exertion by the non-instructed fingers were significantly lower in the elderly in comparison to the young subjects (*p* < 0.001) (Fig. 3). The average delay for all fingers was 100 ± 72 ms and 334 ± 101 ms for elderly and young subjects, respectively. Delay time differed significantly between different fingers (*p* = 0.029), without a significant interaction between age and fingers (*p* = 0.087).

### EMG and force

**Fig. 4:**
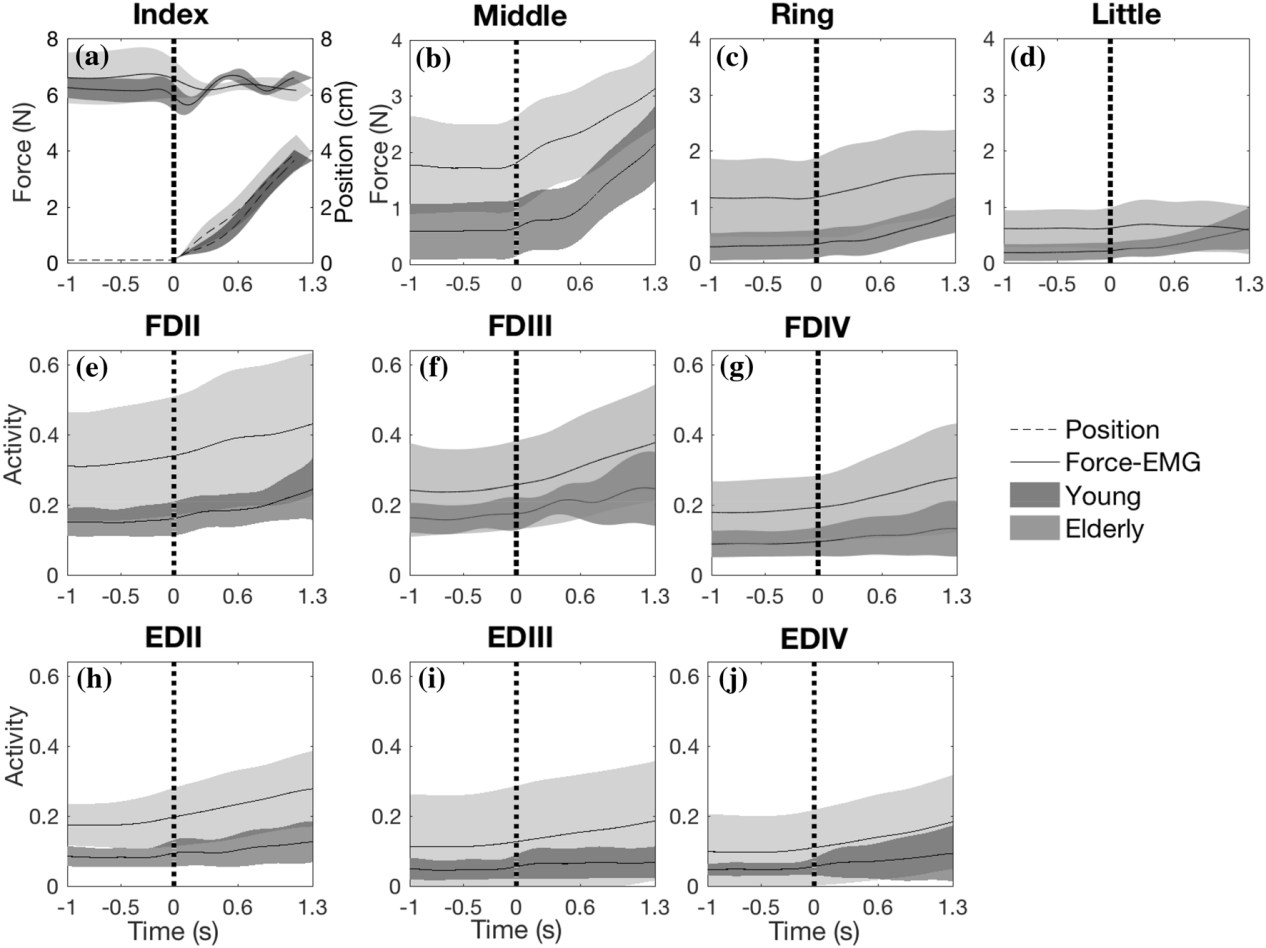
Finger forces (**a**–**d**), FDS EMG activity patterns (**e**–**g**) and ED EMG activity patterns (**h**–**j**) averaged across all elderly subjects (light surface) in comparison with young (dark surface). For index finger (**a**), its position (dashed lines) is also plotted. Vertical dashed line indicates the start of movement

Index finger force was not different between age groups (*p* = 0.754) and did not change significantly in response to the shift from static to dynamic phase (*p* = 0.743; Fig. 4a). There was a significant interaction between age and phase (*p* = 0.029). Post hoc analysis showed that there is no significant difference between phases for young (*p* = 0.166) and elderly (*p* = 0.072). FDS (*p* = 0.001) and ED (*p* = 0.005) EMG activity of the index finger was significantly higher in elderly than in young subjects (Table 1; Fig. 4e, h). FDS (*p* < 0.001) and ED (*p* = 0.007) activity of index finger increased significantly upon flexion. There was a significant interaction between age and phase for ED activity of the index finger (*p* = 0.028), but not for FDS (*p* = 0.143). Post hoc analysis indicated significant differences in ED activity between elderly and young for both the static (*p* = 0.003) and the dynamic phase (*p* = 0.007).

**Table 1:** EMG activity of FDS and ED muscle regions and forces exerted by the fingers for elderly and young subjects during the static and dynamic phases of the task (means ± SD)

Finger	Age group	Force (*N*)			Activity FDS (%MVC)			Activity ED (%MVC)		
		Static phase^†^	Dynamic phase^#^	Change	Static phase^†^	Dynamic phase^#^	Change	Static phase^†^	Dynamic phase^#^	Change
Index	Young	6.2 ± 0.4	6.5 ± 0.2	0.4 ± 0.5	13.9 ± 3.9	18.6 ± 7.7	4.7 ± 6	7.3 ± 2.7	10 ± 6.1	2.7 ± 4.7
	Elderly	6.6 ± 0.9	6.2 ± 0.4	− 0.5 ± 1	32.6 ± 16.8	43.0 ± 20.3	10.4 ± 10	39 ± 29.3	62.4 ± 54.1	23.4 ± 27.1
Middle	Young	0.6 ± 0.5	1.8 ± 0.6	1.2 ± 0.4	16.3 ± 5.8	21.5 ± 15.3	5.2 ± 7.3	4.4 ± 3.2	5.7 ± 4.2	1.3 ± 3
	Elderly	1.7 ± 0.8	3.1 ± 0.7	1.3 ± 0.6	24 ± 10.4	37.1 ± 16.3	13.1 ± 11	10.2 ± 6.9	17.8 ± 10.4	7.6 ± 9.1
Ring	Young	0.3 ± 0.2	0.7 ± 0.3	0.4 ± 0.2	8 ± 3.6	10.9 ± 7.8	2.9 ± 5.3	4.4 ± 2.6	7.5 ± 7.3	3.1 ± 5.3
	Elderly	1.2 ± 0.7	1.6 ± 0.8	0.4 ± 0.6	13.7 ± 5.9	17 ± 3.1	3.4 ± 5.8	9.9 ± 10.7	17.9 ± 13.3	8 ± 4.6
Little	Young	0.2 ± 0.1	0.5 ± 0.3	0.3 ± 0.2	*	*	*	*	*	*
	Elderly	0.6 ± 0.3	0.6 ± 0.4	0.0 ± 0.2	*	*	*	*	*	*

^*^ EMGs of little finger was not measured

^†^ Averaged during the phase

^# ^The value at the end of phase

For forces exerted by the non-instructed fingers, three-way ANOVA (within group factors: finger, static–dynamic phase; between age-group factor: young–elderly) indicated a significant difference between young and elderly (Table 2). Forces across non-instructed fingers were substantially higher in elderly than in young subjects (Fig. 4; Table 1). Force of non-instructed fingers significantly increased upon the flexion of the index finger (Tables 1 and 2). There was no significant interaction between age and phase (Table 2), indicating that the change in non-instructed finger forces in response to the shift from static to dynamic were similar for young and elderly (Table 1). However, a significant interaction between age and finger was found (Table 2). Post-hoc analysis revealed that the non-instructed finger forces were significantly higher in elderly than in young subjects for middle and ring finger (*p* < 0.001) but only close to significance for the little finger (*p* = 0.060).

**Table 2:** *p* and *F* values (*df*1 = factors degree of freedom, *df*2 = errors degree of freedom) of three-way repeated measures ANOVAs [within group factors: finger, static–dynamic phase between group factor: age group (young–elderly)] applied to statistically analyze changes in either finger forces, Enslaving effect, FDS and ED activity of non-instructed fingers

Factors and interactions	Force		Activity FDS		Activity ED		Enslaving effect	
	*F* (*df*1, *df*2)	*p*	*F* (*df*1, *df*2)	*p*	*F* (*df*1, *df*2)	*p*	*F* (*df*1, *df*2)	*p*
Age group	21.77 (1, 18)	< 0.001	9.035 (1, 18)	0.008	18.49 (1, 18)	< 0.001	24.98 (1, 18)	< 0.001
Finger	63.79 (2, 36)	< 0.001	23.51 (1, 18)	< 0.001	0.02 (1, 18)	0.882	66.59 (2, 36)	< 0.001
Static–dynamic phase	76.81 (1, 18)	< 0.001	11.55 (1, 18)	0.003	21.89 (1, 18)	< 0.001	69.61 (1, 18)	< 0.001
Finger × phase	57.8 (2, 36)	< 0.001	11.65 (1, 18)	0.003	0.49 (1, 18)	0.492	60.37 (2, 36)	< 0.001
Age × finger	8.22 (2, 36)	0.001	1.30 (1, 18)	0.269	0.03 (1, 18)	0.859	9.23 (2, 36)	< 0.001
Age × phase	0.13 (1, 18)	0.719	1.34 (1, 18)	0.262	6.79 (1, 18)	0.018	0.44 (1, 18)	0.516
Age × finger × phase	2.8 (2, 36)	0.074	4.53 (1, 18)	0.047	0.18 (1, 18)	0.679	4.91 (2, 36)	0.039

FDS and ED activities of the non-instructed fingers were significantly higher in the elderly than in the young subjects (Tables 1 and 2) (Fig. 4). FDS activity averaged across the non-instructed fingers was around 6 and 11% higher in elderly during the static and dynamic phases, respectively. Similar differences in EMG between young and elderly were found for ED: 6% during static and 10% during the dynamic phase. Three-way ANOVA indicated a significant change in FDS activity due to the shift from static to dynamic phase and a significant interaction between age, finger and static–dynamic phase (Table 2). Two-way ANOVAs (within group factor: finger, between groups: young–elderly) indicated that the change in FDS activity in response to the shift in phase was significantly different between fingers (*p* = 0.003) but not between young and elderly (*p* = 0.262). In addition, a significant interaction between age and finger was found (*p* = 0.047). Post-hoc analysis, however, revealed that the change in FDS activity was not different between young and elderly neither for middle (*p* = 0.138) nor for ring finger (*p* = 0.858). Based on the three-way ANOVA, ED activity was also significantly different between the phases (Table 2). There was also significant interaction between age and static–dynamic phase in ED activity. Post-hoc analysis showed that for the elderly ED activity was higher in the dynamic phase than in the static phase, (*p* < 0.001), but this was not found for the young subjects (*p* = 0.160). These results indicate that ED activity of non-instructed fingers in response to movement of the index finger increased in elderly but not in young subjects.

### Enslaving effect

**Fig. 5:**
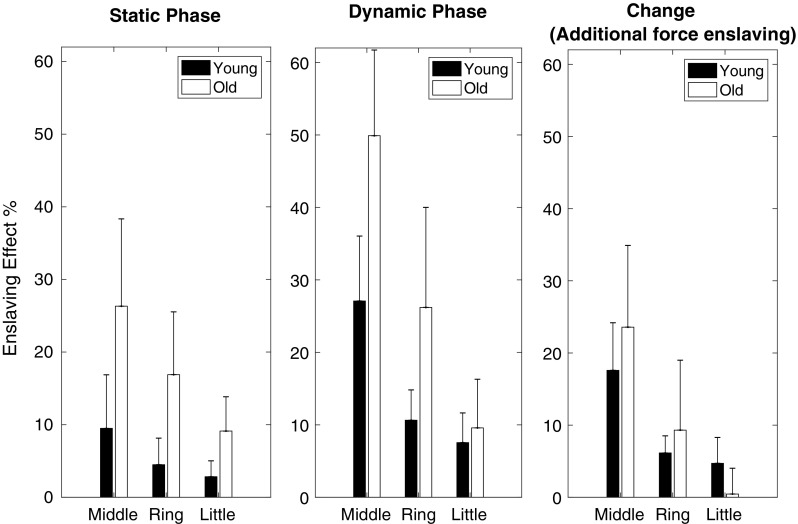
Force enslaving effect (EE) of index movement on non-instructed fingers before the movement (static phase), at the endpoint of the movement (dynamic phase) and the change between them (ΔEE, additional force enslaving) for young and elderly subjects

For the whole task, the enslaving effect (EE) was significantly higher in elderly than young subjects (Table 2; Fig. 5). The difference between young and elderly averaged across all fingers was 12 and 14% during static and dynamic phase, respectively. The EE in the static phase differed significantly from that at the end of the dynamic phase, and there was a significant interaction between age, finger and static–dynamic phase (see Table 2 for statistics). Two-way ANOVAs (within group factor: finger, between groups: young–elderly) indicated the change in enslaving caused by the shift in phase (ΔEE: termed additional force enslaving) was not significantly different between elderly and young subjects (*p* = 0.158). ΔEE was significantly different between fingers (*p* < 0.001) with no significant interaction between finger and age-group (*p* = 0.637). For both young and elderly, the highest and lowest EEs were observed for the middle and little finger, respectively. These results indicate that the magnitude of the enslaving effect was higher in elderly than young, but that both groups responded similarly to index finger flexion.

## Discussion

The aim of this study was to test whether there are age-related changes in force enslaving during dynamic tasks. The major outcomes were: (1) considerably higher non-instructed finger forces in elderly subjects, (2) which started to increase (relative to index finger movement) considerably (more than three times) faster. (3) The response of EMG activities of FDS and ED regions associated with the non-instructed fingers to index finger flexion was different between young and elderly.

The delay of non-instructed finger force in response to index flexion was significantly shorter in elderly than in young subjects (Fig. 2). This could not be explained by differences in movement velocity, because the velocity was not different between age groups. Note that the delay found in elderly (approximately 100 ms) is in agreement with the electromechanical delay (Vos et al. 1990). In young subjects, we previously attributed the longer delay to the incapability of mechanical connections to transmit force at the start of movement (Mirakhorlo et al. 2017). We hypothesized that the mechanical inter-connections between tendons and muscle bellies were slack and not able to transmit force at the beginning of the movement, but they pulled taut and transmit force as a result of relative movement of fingers. The shorter delay found for elderly may be explained by two mechanisms: (1) a shorter slack length of the mechanical linkages in elderly. Effects of aging on slack length were not encountered in the literature; (2) increase in FDS activity of adjacent fingers in elderly, but not in young. We found a significant increase in FDS EMG activity of the non-instructed fingers without any significant interaction between age group and static–dynamic phase (Table 2). However, in our previous study including the young subjects only (Mirakhorlo et al. 2017), no significant changes in EMG activities of non-instructed fingers were found. This suggests that changes in EMG activity of the non-instructed fingers were substantial only in the elderly, possibly explaining the shorter delay.

A higher EE was found in elderly than in young subjects and was accompanied with higher EMG activities of FDS and ED regions of non-instructed fingers. In contrast, all previous studies (Oliveira et al. 2008; Shinohara et al. 2003a, b) reported lower EE indices in elderly. This contradiction might be caused by the differences in the tasks studied. The palm of hand and the forearm were fixed in those studies greatly simplifying the complexity of the task. In the current study, the wrist was not fixed and had to be stabilized by wrist flexors (including FDS) and extensors (including ED). The wrist flexion moment, required as a consequence of exerting force on the fingertip, may be produced partly by FDS and FDP muscles and counter-balanced by ED. Higher activity of all muscle regions of FDS and ED corresponding to the different fingers was probably needed for stabilizing the wrist, which in turn might lead to higher enslaving. Comparing our results with those reported previously (Oliveira et al. 2008) suggests that the enhanced enslaving effect of muscle action required for stabilizing the wrist in the elderly is greater than the enhanced individuation during tasks involving finger control exclusively.

In the present study, the enslaving effect was quantified as the absolute force exerted by the non-instructed finger divided by the force exerted by the instructed finger. Instead of absolute forces, forces normalized to MVC have been used previously to calculate the enslaving effect (Shinohara et al. 2003b). Since the MVC for elderly is lower, the approach to calculate enslaving will influence the extent of the difference between young and elderly (Shinohara et al. 2003b). However, the method for quantifying enslaving will not change the sign of the difference.

This study had several limitations. The EMG activities of only ED and FDS were measured, while several other muscles (such as FDP and the intrinsics) were likely involved also. These muscles can contribute to enslaving limiting our understanding of role of neural factors. However, it was shown that during large movements the intrinsic hand muscles are less active than the extrinsic muscles (Buford et al. 2005) and FDP is active at the similar level as FDS (Ajiboye and Weir 2009; Dai et al. 2001). Using fine wire EMG or a larger electrode array (Van Beek et al. 2018) would have been helpful to detect FDS activities more precisely. However, we used multiple electrodes to cover a broader area to find electrode pairs that as uniquely as possible represented the different muscle regions.

In conclusion, we found higher enslaving effect for elderly than young subjects during both the static and dynamic phase of the studied task. Shorter delay and significant changes in FDS muscle activities in the elderly suggest that the differences in enslaving can at least partly be explained by changes in neuromuscular control.
